# Does presence of metabolic syndrome impact anxiety and depressive disorder screening results in middle aged and elderly individuals? A population based study

**DOI:** 10.1186/s12888-017-1576-8

**Published:** 2018-01-08

**Authors:** Jurate Butnoriene, Vesta Steibliene, Ausra Saudargiene, Adomas Bunevicius

**Affiliations:** 1 Institute of Endocrinology, Lithuanian University of Health Sciences, Eiveniu str. 2, LT-50161 Kaunas, Lithuania; 2 Psychiatry Clinic, Lithuanian University of Health Sciences, Kaunas, Lithuania; 3 Lithuanian University of Health Sciences, Kaunas, Lithuania; 4 Neuroscience Institute, Lithuanian University of Health Sciences, Kaunas, Lithuania

**Keywords:** Depressive disorder, General anxiety disorder, Hospital anxiety and depression scale, Metabolic syndrome, Mini international neuropsychiatric interview, Screening

## Abstract

**Background:**

Depressive and anxiety disorders are common in primary care setting but often remain undiagnosed. Metabolic syndrome (MetS) is also prevalent in the general population and can impair recognition of common mental disorders due to significant co-morbidity and overlap with psychiatric symptoms included in self-reported depression/anxiety screening tools. We investigated if MetS has an impact on the accuracy of current major depressive disorder (MDD) and generalized anxiety disorder (GAD) screening results using the Hospital Anxiety and Depression scale (HADS).

**Methods:**

A total of 1115 (562 men; mean age 62.0 ± 9.6 years) individuals of 45+ years of age were randomly selected from the general population and evaluated for current MetS; depressive and anxiety symptoms (HADS); and current MDD and GAD (Mini International Neuropsychiatric Interview [MINI]).

**Results:**

The MetS was diagnosed in 34.4% of the study participants. Current MDD and GAD were more common in individuals with MetS relative to individuals without MetS (25.3% vs 14.2%, respectively, *p* < 0.001; and 30.2% vs 20.9%, respectively, *p* < 0.001). The ROC analyses demonstrated that optimal thresholds of the HADS-Depression subscale for current MDE were ≥9 in individuals with MetS (sensitivity = 87%, specificity = 73% and PPV = 52%) and ≥8 in individuals without MetS (sensitivity = 81%, specificity = 78% and PPV = 38%). At threshold of ≥9 the HADS-Anxiety subscale demonstrated optimal psychometric properties for current GAD screening in individuals with MetS (sensitivity = 91%, specificity = 85% and PPV = 72%) and without MetS (sensitivity = 84%, specificity = 83% and PPV = 56%).

**Conclusions:**

The HADS is a reliable screening tool for current MDE and GAD in middle aged and elderly population with and without MetS. Optimal thresholds of the HADS-Depression subscale for current MDD is ≥9 for individuals with MetS and ≥8 - without MetS. Optimal threshold of the HADS-Anxiety subscale is ≥9 for current GAD in individuals with and without MetS. The presence of MetS should be considered when interpreting depression screening results.

## Background

Anxiety disorders are the most common psychiatric disorders in primary care patients [1–4]. Patients suffering from anxiety disorders are more likely to seek treatment from primary care provider than from mental health specialist [5]. However, only up to one third of anxiety cases are recognized by primary care providers [3, 6, 7] and only a small proportion of patients receive treatment for anxiety disorders [4, 8, 9]. Generalized anxiety disorder (GAD) is characterized by persistent anxiety and worry, and is the most common anxiety disorder with reported prevalence rates ranging between 2.8% and 8.5% [6, 10]. Untreated GAD causes extreme distress, severe functional impairment [11, 12] significant economic costs [10], and is independently associated with elevated suicide risk [13, 14]. Implementation of effective screening strategies for detection of GAD in primary care setting could increase availability of treatment interventions and consequently contribute towards improved patient outcomes [15–18].

Major depressive disorder (MDD) affects from 5% to 14% of primary care patients [3, 19–21], but only less than 65% of depressed patients are diagnosed with depressive disorders [3, 22, 23] and less than 50% of depressed patients receive antidepressant treatment [23, 24]. Previous clinical practice guidelines did not recommend depression screening in primary care [25–27] due to lack of evidence of benefit of such intervention [28] and concerns for high false positive rate [29]. However, the US Preventive Services Task Force (USPSTF) has recently concluded that depression screening in primary care setting improves identification of depressed patients and treatment availability [30].

Metabolic Syndrome (MetS) encompasses a cluster of cardiovascular disease and type 2 diabetes risk factors [31]. The prevalence of MetS is steadily growing word-wide [32, 33]. Significant co-morbidity and bi-directional association of MetS with depressive and anxiety disorders is well-documented [34–39]. An accumulating body of evidence suggests that biological mechanisms underlying the MetS and MDD can overlap. For example, chronic stress, hyperactivity of hypothalamic-pituitary-adrenal (HPA) axis, noradrenergic dysregulation, inflammatory cytokines and endothelial dysfunction were implicated in both MDD and MetS [40]. Furthermore, behavioral changes attributed to MDD, such as smoking, physical inactivity and sleep disturbances, can also contribute towards development of MetS. On the other hand, metabolic disturbance in MetS patients may contribute to impaired brain functioning and development of GAD, MDD and other affective disorders [41, 42].

These observations indicate the importance of timely identification and adequate management of MDE and GAD among patients with established MetS [31, 36]. However, the MetS is highly co-morbid with other somatic conditions and complaints, which are common in middle aged and elderly population [43]. Symptoms of co-morbid cardiovascular disease, diabetes and obesity (such as fatigue and sleep impairment) can overlap with symptoms included in depression/anxiety self-rating scales leading to impaired recognition of mental disorders and high false positive screening rates due to inclusion of patients reporting symptoms caused by MetS rather than mental disorder. Indeed, previous studies indicated stronger association of MetS with depression evaluated using self-rating scales than structured clinical diagnostic interviews [31, 44]. Therefore, different thresholds of depression/anxiety screening may be needed for patients with co-morbid MetS. The Hospital Anxiety and Depression Scale (HADS) is widely used for screening of depressive and anxiety disorders in medically ill patients [45, 46]. There are no universally accepted thresholds of the HADS as studies documented different optimal thresholds across different somatic disorders [47]. To our knowledge, there are no studies evaluating psychometric properties of the HADS for depressive and anxiety disorder screening purposes as a function of current MetS diagnosis. Therefore, the aim of our study was to investigate if presence of MetS diagnosis has an impact on the accuracy of current MDE and GAD screening using the HADS. We hypothised that due to overlap of MetS-related symptoms with depression, optimal depression (but not anxiety) screening threshold value would be greater in patients with MetS versus patients without MetS.

## Methods

Patient recruitment in this cross-sectional observational cohort study took place from February 2003 until January 2004 at the Primary Health Care Centre (PHCC). Men and women of 45 years old and older were randomly selected from the database of inhabitants registered at the PHCC by using the probability systematic method. Invitation letters were sent to 1624 selected individuals via regular mail. One-thousand one-hundred and twenty (response rate 69%) subjects attended the study visit. Women who did not respond to the study invitation letter were older when compared to women that were studied (*p* < 0.05). Other socio-demographic characteristics were similar between responders and non-responders. However, five individuals were excluded from the analyses because they were not evaluated for the MetS or refused from psychiatric assessment leaving the final sample of 1115 subjects (562 men and 553 women).

All study participants completed a battery of questionnaires that included assessment of socio-demographic characteristics (education, residence, marital status and employment history), and depressive and anxiety symptoms severity (HADS) [46]. Past medical histories and current medication use were evaluated by reviewing medical records. All study participants were evaluated by a trained interviewer for current MDE and GAD using the Mini International Neuropsychiatric Interview or MINI [48].

The study protocol and informed consent procedure were approved by the Regional Bioethics Committee at the Lithuanian University of Health Sciences, Kaunas, Lithuania (2003–01-21 No. 6B/2003). The investigation was carried out in accordance with the Declaration of Helsinki. Each participant gave written informed consent prior to all study procedures.

### Psychiatric evaluation

The HADS [46] is a self-rating questionnaire comprised of two 7-item subscales of anxiety (HADS-A) and depression (HADS-D) that are designed to evaluate respective symptom severity during the preceding 2 weeks. Each HADS item is rated on a 4-point Likert-type scale with total scores on each subscale ranging from 0 to 21, and with higher scores corresponding to greater respective symptom severity [47].

Diagnoses of current MDE and GAD were established using the MINI interview, version 5.0.0 [48]. The MINI interview is a semi-structured diagnostic psychiatric interview designed for evaluation for current psychiatric disorders according to the *Diagnostic and Statistical Manual of Mental Disorders 4th edition, Text revision* (DSM-IV-TR) [49]. The MINI has modular structure pertaining to specific psychiatric diagnoses. For the purpose of the present study we used the MINI modules pertaining to current MDE (module A) and current GAD (module O). The Lithuanian translation of the MINI interview is widely used for research purposes in hospitalized patients [50] and in primary patients [1].

### Assessment of the metabolic syndrome

Participants were asked to fast for at least 12 h prior to the study visit and to collect the first morning urine sample into a special container. During the study visit, fasting blood samples were drawn from an antecubital vein, were centrifuged and stored frozen at −70 °C for measurements of fasting plasma lipid panel and at −20 °C for evaluation of fasting plasma insulin concentration. Serum samples were enzymatically assayed for total cholesterol, high-density lipoprotein (HDL), and triglyceride (TG) concentrations. Low-density lipoprotein (LDL) serum concentration was determined using the Friedwald equation. Fasting glucose (oxidase-phenol aminophenazone method) and insulin (Immunoradiometric method; BioSource INS-IRMA; Belgium) concentrations were also assessed and oral glucose tolerance test [51] was performed in all participants with an exception of diabetic patients. Insulin resistance index was calculated using the Homeostasis Model Assessment (HOMA_IR_) [52]. Urine albumin concentration was determined using immunological semi-quantitative method (Roche Diagnostic; UK). Anthropometric characteristics were measured using standard procedures and included height (in cm), weight (in kg), and waist and hip circumference (in cm). Waist-to-hip ratio (WHR) and body mass index (BMI) were subsequently calculated using standard methods [53, 54]. Blood pressure (BP) was measured using a random-zero mercury sphygmomanometer after the subject sat still for about 5 min.

The diagnosis of the current MetS was established according to the World Health Organization (WHO) criteria that covers impaired insulin resistance or elevated fasting glucose or impaired glucose tolerance or type 2 diabetes mellitus; and any two of the following criteria: (1) arterial hypertension (blood pressure ≥ 140/90 mmHg); (2) current use of antihypertensive medication; (3) elevated TG concentration (≥ 1.7 mmol/l); (4) reduced HDL cholesterol concentration (< 0.9 mmol/l for men or <1.0 mmol/l for women); (5) obesity (BMI > 30 kg/ m^2^, or WHR > 0.9 for men or >0.85 for women); or (6) microalbuminuria [40, 55].

### Statistical analyses

First, we compared socio-demographic characteristics, adverse health behaviors, past medical histories, current medication use, and prevalence of the MINI diagnoses of mental disorders in subjects with MetS versus subjects without MetS by applying the independent sample t-test for continuous factors and Pearson’s chi-squared test for categorical factors.

Next, we sought to identify optimal thresholds of the HADS-D and HADS-A subscales to predict the MINI diagnoses of current MDE and current GAD, respectively, in subjects diagnosed with MetS and in subjects without MetS. We computed sensitivity, specificity, positive predictive value (PPV), negative predictive value (NPV), accuracy and area under the receiver operating characteristic (ROC) curve (AUC), with 95% confidence intervals (CIs). The estimate of the area under the ROC curve was computed nonparametrically. The optimal HADS-D and HADS-A thresholds correctly identifying individuals with MDE and GAD, respectively, were determined as the threshold values that gave the closest to the ideal point on the ROC curve, i.e. that made the resulting binary prediction as close to the perfect predictor as possible [56]. A perfect predictor is represented by a point in the upper left corner in the plot and has 100% sensitivity and 100% specificity. The distance between this optimal point and the ROC curve is estimated using the Euclidean distance. The minimal Euclidean distance indicates the point on the ROC curve with the optimal threshold value.

The SPSS 20.0 for Windows (IBM Corporation, Chicago, IL, USA) and Matlab 7.11 were used for data analysis.

## Results

As presented in Table 1, 34% of the study subjects were diagnosed with current MetS. Subjects with MetS were older (63.7 ± 9.2 years vs 61.1 ± 9.7, respectively; *p* < 0.0001) and were more likely to live in the city (36.5% vs 26.3%, respectively; p < 0.0001), be retired or disabled (70.3% vs 59.5%, respectively; *p* = 0.002), be physically non-active (60.9% vs 48.0%, respectively; p < 0.0001) and do not consume alcohol (14.1% vs 9.7, respectively; *p* = 0.024) when compared to subjects without MetS. Significantly greater prevalence of past medical histories of myocardial infarction (8.6%), stroke (6.8%), cardiovascular disorder (71.6%), type 2 diabetes mellitus (11.7%) and other endocrine disorders (26.8%) was reported in subjects with MetS when compared to patients without MetS. Current medication use was also more common in subjects with MetS.

**Table 1:** Baseline Characteristics in Subjects with MetS and without MetS

	With MetS *n* = 384 (34.4%)	Without MetS *n* = 731 (65.6%)	*P*-value*
Age (years)	63.7 ± 9.2	61.1 ± 9.7	**0.0001**
Gender			0.05
Women	206 (53.6%)	347 (47.5%)	
Men	178 (46.4%)	384 (52.5%)	
Residence			**0.0001**
Rural	244 (63.5%)	539 (73.7%)	
Urban	140 (36.5%)	192 (26.3%)	
Education			0.219
High school or lower	193 (50.3%)	341 (46.6%)	
Graduated from high school	150 (39.1%)	324 (44.3%)	
University degree	41 (10.7%)	66 (9.0%)	
Marital status			0.152
Currently married	260 (67.7%)	531 (72.6%)	
Never married	20 (5.2%)	40 (5.5%)	
Divorced or widower	104 (27.1%)	160 (21.9%)	
Employment status			**0.002**
Currently employed	71 (18.5%)	188 (25.7%)	
Currently unemployed	43 (11.2%)	108 (14.8%)	
Retired/disabled	270 (70.3%)	435 (59.5%)	
Current smoking			0.329
Non-smokers	266 (69.3%)	478 (65.4%)	
Smokes 1–9 cigarettes/day	19 (4.9%)	54 (7.4%)	
Smokes 10–19 cigarettes/day	38 (9.9%)	69 (9.4%)	
Smokes ≥20 cigarettes/day	61 (15.9%)	130 (17.8%)	
Alcohol consumption			**0.024**
Non-consumers	54 (14.1%)	71 (9.7%)	
Several times/year	210 (54.7%)	384 (52.5%)	
Monthly or more	120 (31.2%)	276 (37.8%)	
Physical activity ^a^			**0.0001**
Several times/month or more	75 (19.5%)	124 (17.0%)	
Several times/year	75 (19.5%)	256 (35.0%)	
Non-active	234 (60.9%)	351 (48.0%)	
Past medical histories			
Myocardial infarction	33 (8.6%)	29 (4.0%)	**0.001**
Stroke	26 (6.8%)	28 (3.8%)	**0.030**
Cardiovascular disorders	275 (71.6%)	388 (53.1%)	**0.0001**
Respiratory disorders	138 (35.9%)	232 (31.7%)	0.157
Gastrointestinal disorders	131 (34.1%)	241 (33.0%)	0.70
Musculoskeletal disorders	204 (53.1%)	387 (52.9%)	0.953
Neurological disorders	86 (22.4%)	175 (23.9%)	0.563
Genitourinary tract disorders	110 (28.6%)	187 (25.6%)	0.271
Type 2 diabetes mellitus	45 (11.7%)	8 (1.1%)	**0.0001**
Other endocrine disorders	103 (26.8%)	118 (16.1%)	**0.0001**
Other diseases	135 (35.2%)	231 (31.6%)	0.230
Current medication use			
ACE inhibitors	144 (37.5%)	97 (13.3%)	**0.0001**
Beta-blockers	41 (10.7%)	29 (4.0%)	**0.0001**
Calcium channel blockers	43 (11.2%)	26 (3.6%)	**0.0001**
Other medication	98 (25.5%)	107 (14.6%)	**0.0001**
Current MINI mental disorders			
Generalized Anxiety Disorder	116 (30.2%)	153 (20.9%)	**0.001**
Major Depressive Episode	97 (25.3%)	104 (14.2%)	**0.0001**

Values are expressed as no. of subjects (%) or mean ± SD

*ACE* angiotensin-converting-enzyme inhibitor, *MINI* Mini International Neuropsychiatric Interview, *SD* standard deviation

^a^At least 30 min of physical exercise per day

* *p* value were calculated using independent sample t-test or Pearson’s chi-squared test

In **bold** *p* values <0.05

Prevalence rates of current MDE and GAD were significantly higher among subjects with MetS in comparison to subjects without MetS (25.3% vs. 14.2%, respectively; *p* < 0.001; and 30.2% vs 20.9%, respectively; *p* < 0.0001).

**Fig. 1:**
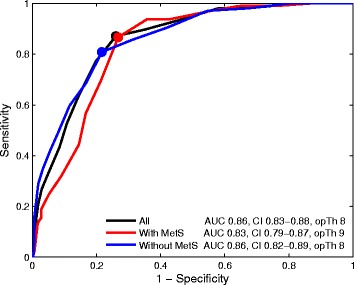
The areas under the ROC curves and the optimal thresholds of the HADS-D for current MDE in all study participants, subjects with MetS and subjects without MetS

The ROC analyses showed that AUCs of the HADS-D for current MDE in subjects with MetS and without MetS were at levels of 0.83 (95% CI: 0.79–0.87) and 0.86 (95% CI: 0.82–0.89), respectively. Psychometric properties of the HADS-D at different thresholds for current MDE in individuals with and without MetS are presented in Table 2. Optimal thresholds of the HADS-D for current MDE were ≥9 in subjects with MetS (sensitivity = 87%, specificity = 73% and PPV = 52%) and ≥8 in subjects without MetS (sensitivity = 81%, specificity = 78% and PPV = 38%). In the total sample, the HADS-D at threshold of ≥8 demonstrated optimal psychometric properties for current MDE screening (sensitivity = 87%, specificity = 74% and PPV = 42%) (Fig. 1).

**Table 2:** Receiver-operating Characteristics for Current Major Depressive Episode using Different Thresholds of the Depression Subscale of the Hospital Anxiety and Depression Scale (HADS-D)

Thresholds	*n* (%)	Sensitivity, % (95% CI)	Specificity, % (95% CI)	PPV, % (95% CI)	NPV, % (95% CI)	Accuracy, % (95% CI)	AUC (95% CI)
All study participants, *n* = 1115							
≥ 6	610 (55)	94 (89–96)	54 (51–57)	31 (27–35)	97 (96–99)	61 (58–64)	0.86 (0.83–0.88)
≥ 7	499 (45)	90 (84–93)	65 (62–68)	36 (32–40)	97 (95–98)	70 (67–72)	
**≥ 8**	**414 (37)**	**87 (81–91)**	**74 (71–77)**	**42 (38–47)**	**96 (95–98)**	**76 (74–79)**	
≥ 9	336 (30)	77 (71–83)	80 (77–83)	46 (41–52)	94 (92–96)	80 (77–82)	
≥ 10	263 (24)	64 (57–71)	85 (83–88)	49 (43–55)	92 (89–93)	81 (79–84)	
With Metabolic syndrome only (WHO definition), *n* = 384							
≥ 6	252 (66)	97 (91–100)	45 (39–51)	37 (31–44)	98 (93–100)	58 (53–63)	0.83 (0.79–0.87)
≥ 7	215 (56)	94 (86–98)	57 (51–63)	42 (36–49)	96 (92–99)	66 (61–71)	
≥ 8	194 (51)	94 (86–98)	64 (58–70)	47 (40–54)	97 (93–99)	72 (67–76)	
**≥ 9**	**161 (42)**	**87 (78–93)**	**73 (68–78)**	**52 (44–60)**	**94 (90–97)**	**77 (72–81)**	
≥ 10	128 (33)	69 (59–78)	79 (73–83)	52 (43–61)	88 (84–92)	88 (84–92)	
Without Metabolic syndrome only (WHO definition), *n* = 731							
≥ 6	358 (49)	90 (83–95)	58 (54–62)	26 (22–31)	97 (95–99)	63 (59–66)	0.86 (0.82–0.89)
≥ 7	284 (39)	86 (77–92)	69 (65–72)	31 (26–37)	97 (94–98)	71 (68–75)	
**≥ 8**	**220 (30)**	**81 (72–88)**	**78 (75–81)**	**38 (32–45)**	**96 (94–98)**	**79 (75–82)**	
≥ 9	175 (24)	68 (58–77)	83 (80–86)	41 (33–48)	94 (92–96)	81 (78–84)	
≥ 10	135 (18)	60 (50–69)	88 (86–91)	46 (37–55)	93 (91–95)	84 (81–87)	

*PPV* positive predictive value, *NPV* negative predictive value, *AUC* area under the receiver operating curve, *CI* confidence interval

Optimal thresholds in **bold**

**Fig. 2:**
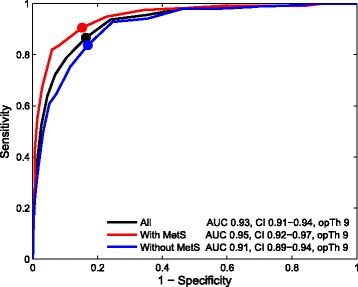
The areas under the ROC curves and the optimal thresholds of the HADS-A for current MDE in all study participants, subjects with MetS and subjects without MetS

AUCs of the HADS-A for current GAD were 0.95 (95% CI: 0.92–0.97) in subjects with MetS and 0.91 (95% CI: 0.89–0.94) in subjects without MetS. Psychometric properties of the HADS-A at different thresholds as a function of presence of the MetS for screening of current GAD are presented in Table 3. We found that at threshold of ≥9 the HADS-A demonstrated optimal psychometric properties for current GAD in subjects with MetS (sensitivity = 91%, specificity = 85% and PPV = 72%) and without MetS (sensitivity = 84%, specificity = 83% and PPV = 56%) (Fig. 2).

**Table 3:** Receiver-operating Characteristics for Generalized Anxiety Disorder using Different Thresholds of the Anxiety Subscale of the Hospital Anxiety and Depression Scale (HADS-A)

Thresholds	*n* (%)	Sensitivity, % (95% CI)	Specificity, % (95% CI)	PPV, % (95% CI)	NPV, % (95% CI)	Accuracy, % (95% CI)	AUC (95% CI)
All study participants, *n* = 1115							
≥ 6	661 (59)	98 (95–99)	53 (50–56)	40 (36–44)	99 (97–100)	64 (61–67)	0.93 (0.91–0.94)
≥ 7	554 (50)	96 (92–98)	65 (62–68)	46 (42–51)	98 (96–99)	72 (70–75)	
≥ 8	456 (41)	94 (90–96)	76 (73–79)	55 (51–60)	97 (96–98)	80 (78–82)	
**≥ 9**	**373 (33)**	**87 (82–90)**	**84 (81–86)**	**63 (57–67)**	**95 (93–97)**	**84 (82–86)**	
≥ 10	301 (27)	79 (73–83)	90 (87–91)	70 (65–76)	93 (91–95)	87 (85–89)	
With Metabolic syndrome only (WHO definition), *n* = 384							
≥ 6	242 (63)	98 (93–100)	52 (46–58)	47 (41–54)	99 (95–100)	66 (61–71)	0.95 (0.92–0.97)
≥ 7	205 (53)	97 (92–100)	66 (60–71)	55 (48–62)	98 (95–100)	75 (71–79)	
≥ 8	171 (45)	95 (89–98)	77 (72–82)	64 (57–71)	97 (94–99)	83 (78–86)	
**≥ 9**	**146 (38)**	**91 (83–95)**	**85 (80–89)**	**72 (64–79)**	**95 (92–98)**	**86 (83–90)**	
≥ 10	119 (31)	84 (75–90)	92 (88–95)	82 (73–88)	93 (89–96)	89 (86–92)	
Without Metabolic syndrome only (WHO definition), *n* = 731							
≥ 6	419 (57)	98 (94–100)	53 (49–58)	36 (31–41)	99 (97–100)	63 (59–66)	0.91 (0.89–0.94)
≥ 7	349 (48)	94 (89–97)	65 (60–68)	41 (36–47)	98 (95–99)	71 (67–74)	
≥ 8	285 (39)	93 (87–96)	75 (72–79)	50 (44–56)	98 (95–99)	79 (76–82)	
**≥ 9**	**227 (31)**	**84 (77–89)**	**83 (80–86)**	**56 (50–63)**	**95 (93–97)**	**83 (80–86)**	
≥ 10	182 (25)	75 (67–82)	88 (85–91)	63 (56–70)	93 (91–95)	86 (83–88)	

*PPV* positive predictive value, *NPV* negative predictive value, *AUC* area under the receiver operating curve, *CI* confidence interval

Optimal thresholds in **bold**

## Discussion

We found that the HADS is a reliable screening tool for current MDE and GAD irrespectively of the presence of the MetS. Optimal thresholds of the HADS-D for current MDE were ≥9 for subjects with MetS and ≥8 for subjects without MetS. Optimal threshold of the HADS-A for current GAD was ≥9 in subjects with and without MetS. At optimal thresholds the HADS-D and HADS-A demonstrated adequate sensitivity, specificity and PPV for current MDE and GAD, respectively, in subjects with and without MetS.

Optimal thresholds of the HADS-D for current MDE screening was higher in participants with MetS, relative to participants without MetS. These findings suggest that somatic symptoms attributed to conditions co-morbid with MetS can overlap with depressive symptoms and consequentially inflate the total HADS-D score. For example, central obesity is among the cardinal MetS features that can cause symptoms similar to depressive disorders, such as general tiredness, fatigue and reduced physical activity. Consequentially patients with MetS can rate the HADS-D item “feeling of slowed down” higher due to obesity rather than depression. From social perspective, obese patients can have lower self-esteem resulting in decreased enjoyment in the social activities [57] and therefore rate higher the HADS-D items “the ability still enjoy the things, were used to enjoy” and “the loss of interest in personal appearance”. Similar findings were reported in a systematic review and meta-analysis by Pan et al., [31], showing that self-reported depression instruments among subjects with MetS allow inclusion of patients who do not meet the DSM diagnostic criteria for current MDD resulting in increased false-positive rate. Our findings suggest that higher optimal HADS-D threshold (≥9) for current MDE screening purposes should be used in subjects with MetS, as this approach would reduce the likelihood of false positive screening results by excluding individuals with MetS-related symptoms mimicking clinical depressive symptoms. Optimization of false-positive depression screening results by using greater threshold could potentially reduce delivery of unnecessary treatment interventions and reduce burden on available healthcare resources.

In our cohort, patients diagnosed with MetS were slightly older, had more somatic co-morbidities and used more prescription medication relative to non-MetS patients, suggesting that greater somatic symptom burden among individuals with MetS that can mimic depressive symptoms and impact depression screening. Indeed, optimal HADS-D threshold of ≥9 for current MDD screening were previously reported in other somatic patient populations. For example, optimal HADS-D threshold of ≥9 (sensitivity 0.846 and specificity 0.903) was found in the general medical ward patients [58]; and ≥10 (sensitivity 0.77 and specificity 0.82) in patients with chronic fatigue syndrome [59] and general medical out-patients with unexplained somatic symptoms (sensitivity 0.56 and specificity 0.92) [60]. Also, optimal threshold of the HADS-D at ≥11 was reported in patients with advanced breast cancer (sensitivity 0.75 and specificity 0.75) [61], Hodgkin’s lymphoma and non-Hodgkin lymphoma (0.84 sensitivity and 0.66 specificity) [62]; and in mixed sample of breast cancer, head and neck cancers, and lymphoma outpatients (0.43 sensitivity and 0.96 specificity) [63]. These findings suggest that MDD screening results using the HADS-D should be interpreted with caution in the context of patients’ functional status and other somatic co-morbidities, including the MetS.

We found that histories of cardiovascular disorder (CVD) and type 2 diabetes were more common in subjects with MetS relative to subjects without MetS. However, previous studies in patients with established CVD reported lower optimal HADS-D thresholds for screening of depressive disorders relative to our study. Optimal HADS-D thresholds was ≥4 (sensitivity 0.864, a specificity 0.788) in out-patients with chronic heart failure [64]; ≥5 (sensitivity 0.77%, specificity 0.69 and PPD 0.23%) in CVD patients attending cardiac rehabilitation [65]; and ≥8 in stable coronary heart disease (CHD) patients [66]. These findings suggest that in MetS population, non-CVD related symptoms other comorbid non-CVD disorders can account for inflation self-reported depressive symptom severity. In patients with diabetes, glucose metabolism impairment can also account for greater self-reported depressive symptom severity because it is known that greater self-perceived depression among patients with MetS is associated with larger waist circumference among women and with elevated plasma glucose concentration among men [67]. Studies examining potential impact of co-morbid diabetes on depression screening results should be attempted.

In the present cohort, patients with MetS were more likely to be retired/ disabled, live in urban area, report less physical activity and greater alcohol consumption. These socioeconomic, environmental and behavioral markers were also linked to depressive disorders [68] indicating that common mental disorders and MetS can share environmental risk factors. Better understanding of common features underlying common mental disorders and MetS could potentially help to identify vulnerable populations, and to develop more accurate recognition strategies and effective interventions leading to reduced global burden of the two disorders.

In our study, the optimal HADS-D cutoff score of the current MDE was at level of ≥8 in subjects without MetS and in the total sample of the study participants. Lower optimal HADS-D thresholds of ≥6 (sensitivity 0.66; specificity 0.97) and ≥7 (sensitivity 0.8; specificity 0.69) for current MDD screening were previously reported by other studies in primary care patients [58]. These findings can be explained that we considered only middle aged and elderly patients as opposed to consecutive primary care patients included in the latter two studies. Elderly patients are expected to have more co–morbidities that can interfere with depression screening. Our findings suggest that HADS-D cutoff score of ≥8 should be used for current MDE screening purposes in middle aged and elderly patients.

We found that the optimal threshold (≥9) of the HADS-A was the same for screening for current GAD in subjects with and without MetS. These findings suggest that GAD symptoms do not overlap with symptoms of MetS and MetS related co–morbidities. The same optimal threshold of the HADS-A at a level of ≥9 for current GAD [45, 69] was reported by other groups in primary care populations. Screening for current GAD should be considered in patients with MetS, because GAD and MetS can increase cardiovascular mortality risk in women independently from each other [70]. Unrecognized and untreated GAD can significantly impair quality of life and level of functioning, and impose burden to societies [71].

Both HADS subscales had high sensitivity for MDD and GAD screening; however, positive predictive values were low. These findings indicate high-false positive rate of current MDE and GAD screening using the HADS. Therefore, all patients screened positive using the HADS scale should be referred for psychiatric consultation prior to initiation of treatment interventions. Further studies investigating optimal mental disorders screening algorithms in primary care setting are encouraged.

Limitations of our study should be acknowledged. Inclusion of patients from predominantly rural area can put our results at risk for selection bias and limit generalizability to urban population. Also, only middle aged and elderly patients were invited in the study, therefore our results cannot be generalized to younger patients.

There is a debate in literature about the validity of the MetS definition criteria [72, 73]. We used the MetS definition proposed by the WHO that considers insulin resistance as the cardinal MetS feature and it is among the most widely studied MetS diagnostic criteria. However, studies examining potential impact of the MetS diagnosed using other commonly used sets of diagnostic criteria for mental disorder screening can be attempted. We evaluated psychometric properties of one scale (the HADS) that is commonly used for screening purposes in clinical setting. Psychometric properties of other commonly used depression screening scales, such as the Patient Health Questionnaire, remain to be investigated in patients with MetS. On the other hand, strengths of our study include large and representative sample of middle aged and elderly general population, use of validated diagnostic psychiatric interview and the use of well-established WHO criteria for diagnosis of current MetS.

## Conclusions

In conclusion, the HADS has adequate psychometric properties for current MDE and GAD screening purposes in middle aged and elderly patients with and without MetS. For current MDD screening, the HADS-D threshold of ≥9 should be used in patients with MetS and ≥8 in patients without MetS with acceptable sensitivity (≥87%) and specificity (≥73%). For current GAD screening the HADS-A threshold of ≥9 has adequate psychometric (sensitivity ≥87% and specificity ≥84%) in patients with and without MetS. Further studies investigating optimal mental disorder screening strategies in primary care settings are encouraged.

## Abbreviations

ACE: Angiotensin-converting-enzyme inhibitor
AUC: Area under the receiver operating curve
BMI: Body mass index
BP: Blood pressure
CHD: Coronary heart disease
CI: Confidence interval
CVD: Cardiovascular disorders
DSM-IV-TR: Diagnostic and statistical manual of mental disorders 4th edition, text revision
GAD: Generalized anxiety disorders
HADS: Hospital anxiety and depression scale
HADS-A: Hospital anxiety and depression scale - anxiety subscale
HADS-D: Hospital anxiety and depression scale - depression subscale
HDL: High-density lipoprotein
HOMA_IR_: Homeostasis model assessment
LDL: Low-density lipoprotein
MDD: Major depressive disorder
MDE: Major depressive episode
MetS: Metabolic syndrome
MINI: Mini International neuropsychiatric interview
NPV: Negative predictive value
PHCC: Primary health care centre
PPV: Positive predictive value
ROC: The receiver operating characteristic
SD: Standard deviation
TG: Triglyceride
USPSTF: US preventive services task force
WHO: World health organization
WHR: Waist-to-hip ratio
